# Ethylene Glycol Dicyclopentenyl (Meth)Acrylate Homo and Block Copolymers via Nitroxide Mediated Polymerization

**DOI:** 10.3390/ma12091547

**Published:** 2019-05-11

**Authors:** Alexandre Maupu, Yara Kanawati, Adrien Métafiot, Milan Maric

**Affiliations:** Department of Chemical Engineering, Centre québécois sur les matériaux fonctionnels/Quebec Centre for Advanced Materials (CQMF/QCAM), Centre de recherche sur les systèmes polymères et composites à haute performance (CREPEC), McGill Institute of Advanced Materials (MIAM), McGill University, Montreal, QC H3A 0C5, Canada; alexandre.maupu@mail.mcgill.ca (A.M.); yara.kanawati@mail.mcgill.ca (Y.K.); adrien.metafiot2@mcgill.ca (A.M.)

**Keywords:** nitroxide mediated polymerization, copolymerization, block copolymers

## Abstract

Nitroxide-mediated polymerization (NMP), (homo and block copolymerization with styrene (S) and butyl methacrylate/S) of ethylene glycol dicyclopentenyl ether (meth)acrylates (EGDEA and EGDEMA) was studied using BlocBuilder alkoxyamines. EGDEA homopolymerization was not well-controlled, independent of temperature (90–120 °C), or additional free nitroxide (0–10 mol%) used. Number average molecular weights (*M_n_*) achieved for poly(EGDEA) were 4.0–9.5 kg mol^−1^ and were accompanied by high dispersity (*Ð* = *M_w_/M_n_* = 1.62–2.09). Re-initiation and chain extension of the poly(EGDEA) chains with styrene (S) indicated some block copolymer formation, but a high fraction of chains were terminated irreversibly. EGDEA-*stat*-S statistical copolymerizations with a low mol fraction S in initial feed, *f_S_*_,0_ = 0.05, were slightly better controlled compared to poly(EGDEA) homopolymerizations (*Ð* was reduced to 1.44 compared to 1.62 at similar conditions). EGDEMA, in contrast, was successfully polymerized using a small fraction of S (*f_S_*_,0_ ~ 10 mol%) to high conversion (72%) to form well-defined EGDEMA-rich random copolymer (molar composition = *F_EGDEMA_* = 0.87) of *M_n_* = 14.3 kg mol^−1^ and *Ð* = 1.38. EGDEMA-rich compositions were also polymerized with the unimolecular succinimidyl ester form of BlocBuilder initiator, NHS-BlocBuilder with similar results, although *Ðs* were higher ~1.6. Chain extensions resulted in monomodal shifts to higher molecular weights, indicating good chain end fidelity.

## 1. Introduction

Ethylene glycol dicyclopentenyl ether acrylate (EGDEA) and the related ethylene glycol dicyclopentenyl ether methacrylate (EGDEMA) are imparted into polymers useful as reactive coalescents that aid film formation in coatings [1,2], reactive diluents to reduce exotherms in bone cement formulations [3], and anti-bacterial films and coatings without resorting to quaternary ammonium functionalities or metallic nanoparticles [4,5,6,7]. Additionally, EGDEA and EGDEMA also possess norbornene functionalities that open possibilities for orthogonal functionality, allowing modifications via thiol-ene click chemistry, for example [8,9,10,11]. Initially, these two particular monomers were polymerized by conventional radical polymerization using photoinitiation (cross-linking) [4] and with peroxide or nitrile-based initiators [3]. In these cases, EGDEA and EGDEMA-containing polymers do not have the control over microstructure and molecular weight distribution that would impart additional properties. For example, low viscosity coating solutions can result from narrow molecular weight distribution resins [12,13,14], while block copolymers can provide the possibility of self-assembly with domains containing specific functionality [15]. Controlled radical polymerization, more succinctly termed as reversible de-activation radical polymerization (RDRP), has emerged as the route to make such structured polymers, which previously was the domain exclusively of living polymerizations such as ionic or group transfer polymerizations [16]. Methods such as atom transfer radical polymerization (ATRP) [17,18], reversible addition fragmentation transfer polymerization (RAFT) [19] and nitroxide mediated polymerization (NMP) [20] are now commonly used to make such controlled microstructure polymers. Using ATRP, EGDEMA was homopolymerized to form end-blocks with poly(2-ethylhexyl acrylate) center blocks in ABA triblock copolymers where the end-blocks were modified by thiol-ene click reactions to improve the adhesion strength [11]. There have been no other reports using EGDEA or EGDEMA in other RDRP processes to the best of our knowledge.

NMP is attractive due to its relative simplicity in terms of initiation and the limited post-polymerization work-up required (i.e., no catalyst residues or discolouration) [21,22,23]. Traditionally, acrylate homopolymerization by NMP was limited due to the high equilibrium constants between dormant and active chains associated with acrylates [24] and methacrylates [25,26] with first generation TEMPO-type nitroxides as mediators. This means that the active state is comparatively more probable, thus leading to a higher accumulation of radical species that result in irreversible termination [25]. In some cases, however photo-controlled radical polymerization with TEMPO for methyl methacrylate homopolymerizations was successful using 4-hydroxy TEMPO with a photo-acid generator such as bis(alkylphenyl)iodonium hexafluorophosphate (BAI) [27]. Various bulk, solution and dispersion polymerizations were demonstrated using such combinations at ambient temperatures, resulting in fairly high degrees of polymerizations (M_n_ up to 30 kg mol^−1^) with dispersities approaching 1.5. With so-called second-generation initiators such as the BlocBuilder/SG1 or TIPNO (2,2,5-Trimethyl-4-phenyl-3-azahexane-3-nitroxide) families, acrylate homopolymerization, and methacrylate polymerizations with a small concentration of controlling co-monomer (~5–10 mol%) were enabled [25]. More recently, methacrylate homopolymerizations by NMP have been developed using in situ nitroxide formation [28], a DPIAO unimolecular initiator (2,2-diphenyl-3-phenylimino-2,3-dihydroindol-1-yloxyl nitroxide) where the poly(methyl methacrylate) macroinitiator could not re-initiate cleanly a second block of monomer such as styrene [29] while a succeeding design was successful in crossing over to the styrenic block [30]. More recently, Dispolreg 007 nitroxide initiator was capable of homopolymerizing methacrylates, and cross-over to a styrenic block was cleanly done [31,32,33].

In this study, we used BlocBuilder, a commercially available unimolecular initiator, to control EGDEA and EGDEMA homo, statistical (at low co-monomer compositions to aid in control of polymerization) and block copolymerizations to test chain end fidelity. We first examined the kinetics of EGDEA homopolymerization at different temperatures as well as additional free SG1 nitroxide. Chain extension experiments with a second monomer, styrene, were then performed to evaluate the chain-end fidelity of the EGDEA homopolymers. We next examined EGDEMA controlled polymerizations using styrene as a controlling co-monomer and offer a starting point towards further applications of these functional monomers via RDRP processes.

## 2. Materials and Methods 

Dioxane (≥99%) was obtained from Sigma Aldrich (Milwaukee, WI, USA) and used as received. For solvents and precipitants, methanol (≥99.8%) and tetrahydrofuran (THF, >99%, HPLC grade) were acquired from Fisher Scientific (Whitby, Canada) and used as received while deuterated chloroform (CDCl_3_, >99%) was obtained from Cambridge Isotopes Laboratory (Tewksbury, MA, USA) and was also used as received. Ethylene glycol dicylopentenyl ether acrylate (EGDEA, inhibited by 700 ppm MEHQ), ethylene glycol dicyclopentenyl ether methacrylate (EGDEMA, inhibited by 100 ppm hydroquinone), styrene (S) and butyl methacrylate (BMA) were obtained from Sigma-Aldrich. To remove the inhibitors, calcium hydride (90–95%, reagent grade), basic alumina (Brockmann, type 1, 150 mesh) were used and also obtained from Sigma-Aldrich. All monomers (EGDEA, EGDEMA, S and BMA) were purified by passage through a column of 5 wt.% calcium hydride relative to basic alumina and stored in a refrigerator in a sealed flask under a head of nitrogen until required. 2-([tert-butyl[1-(diethoxyphosphoryl)-2,2-dimethylpropyl]amino]oxy)-2-methylpropionic acid, also known as BlocBuilder (99%), was obtained from Arkema (King of Prussia, PA, USA) and used without further purification. [tert-butyl[1-(diethoxyphosphoryl)-2,2-dimethylpropyl]amino] nitroxide, also known as SG1 (>85%) was kindly donated by Noah Macy of Arkema and also used as received. Succinimidyl ester terminated BlocBuilder was synthesized following a procedure from the literature [34]. Poly(methyl methacrylate) (PMMA) narrow molecular weight distribution standards (875–1,677,000 g mol^−1^) were purchased from Varian Canada (Mississauga, Canada).

### 2.1. Homopolymerization of EGDEA

**Figure 1:**
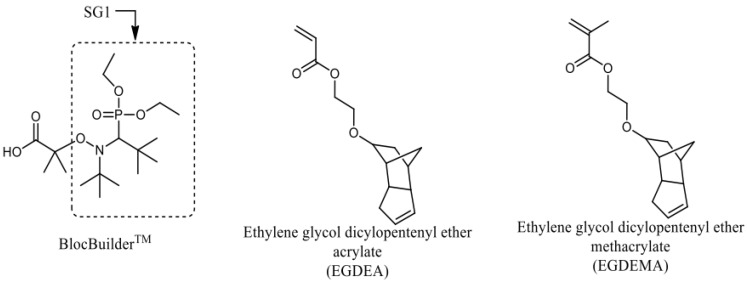
Structures of BlocBuilder, SG1 free nitroxide and ethylene glycol dicyclopentenyl ether (meth)acrylate (EGDEA and EGDEMA).

All EGDEA homopolymerizations were performed in a 25 mL three-neck round-bottom flask equipped with a reflux condenser, thermal well and magnetic stir bar. The amounts of initiator and monomer were calculated to achieve a target molecular weight (M_n,target_), at 100% conversion) of 20 kg mol^−1^. The % molar ratio of free SG1 nitroxide relative to BlocBuilder^TM^ was varied from 0%–10%; 1,4-dioxane (monomer concentration 50 wt.%) was added to dissolve the monomer and keep the viscosity low. The polymerization temperatures were varied from 90–120 °C. Figure 1 shows the structures of BlocBuilder and SG1 along with the EGDEA and EGDEMA monomers. A summary of the experimental conditions for all homopolymerizations of EGDEA are listed in Table 1. Experiment [E-T90] listed in Table 1 is provided as an example. A mixture of BlocBuilder (0.1072 g, 0.281 mmol), free SG1 (0.0044 g, 0.015 mmol), EGDEA (5.4518 g, 21.955 mmol), and 1,4-dioxane (5.4646 g, 62.020 mmol) was added to the reactor with a magnetic stir bar. The mixture was purged with purge for 30 min at room temperature with stirring. Theh, the contents were heated at a rate of 10 °C min^−1^. When the temperature reached 90 °C, the initial start of the reaction (t = 0 min) was assumed. Samples were periodically withdrawn by syringe. A small portion of the withdrawn sample was used for determining monomer conversion as a function of time via ^1^H NMR. The remaining portion of the sample was precipitated in excess cold methanol to remove any unreacted monomers before analysis by gel permeation chromatography. The polymerization was stopped at 4.5 h and the final homopolymer was recovered by using precipitation into methanol followed by drying overnight in a vacuum oven at 25 °C. This polymer was recovered with a yield of 1.2551 g (yield = 23%, overall conversion determined by ^1^H NMR = 22%, number-average molecular weight *M_n_* = 4.0 kg mol^−1^ and dispersity *Ð* = 1.62). Molecular weight and dispersity of the samples and polymer products were measured using gel permeation chromatography (GPC) (Waters Breeze) and were relative to PMMA standards in THF at 40 °C. Details concerning the polymer characterization by ^1^H NMR and gel permeation chromatography will be discussed in the upcoming sections.

### 2.2. Synthesis of Poly(EGDEA/S) and Poly(EGDEMA/S) Statistical Copolymers

#### 2.2.1. EGDEA/S and EGDEMA/S Statistical Copolymers with BlocBuilder/SG1

The EGDEA and EGDEMA statistical copolymerizations with styrene (S) were performed using a 50 wt.% dioxane solution where the amount of BlocBuilder was calculated to give an approximate target molecular weight of 20 kg mol^−1^. Similar reactors were used as that for the EGDEA homopolymerizations. The polymerization temperature was set at 90 °C. The ratio of free nitroxide to initiator was 5 mol% SG1 additional free nitroxide relative to BlocBuilder (this is termed *r*) and 5–15 mol% styrene in the initial monomer mixture, used as a controlling comonomer, was added. A formulation with the EGDEA copolymerization at 90 °C with *r* = 0.05 and 5 mol% styrene is given as an example (Expt. ID: EGDEA/S, Table 2): BlocBuilder (0.1013 g, 0.265 mmol), SG1 (0.0040 g, 0.013 mmol), EGDEA (5.4779 g, 22.060 mmol), styrene (0.1232 g, 1.183 mmol), and 1,4-dioxane (5.4732 g, 62.118 mmol) were added to the reactor prior to being sealed with a rubber septum. A summary of all other formulations of the EGDEA/S copolymerization and EGDEMA/S random copolymerization can be found in Table 2. The mixture was purged with nitrogen for 30 min at room temperature prior to being heated at a rate 10 °C min^−1^. The start of the reaction (*t* = 0 min) was assumed when the temperature reached 90 °C. Samples were periodically withdrawn by syringe and precipitated in an excess of cold methanol to remove any remaining unreacted monomers. The final polymer was recovered after similar precipitation followed by drying in the vacuum oven for 6 h at 25 °C. For the specific example cited, the polymerization was stopped after 5.3 h and the final homopolymer was characterized by a yield of 0.5193 g (17.5% overall conversion according to ^1^H NMR) with *M_n_* = 3.6 kg mol^−1^ and *Ð* = *M_w_/M_n_* = 1.44 according to GPC using poly(methyl methacrylate) standards in THF at 40 °C.

#### 2.2.2. EGDEMA/S Statistical Copolymers Using NHS-BlocBuilder

The same reactor set-up was employed for the set of experiments using NHS-BlocBuilder instead of the BlocBuilder/SG1 combination for the nitroxide. A list of all formulations for this set of experiments is provided in Table 3. A typical EGDEMA/S polymerization with NHS-BlocBuilder is given as an example (Expt. ID EGDEMA/S-N-15-90 from Table 3). 4.7912 g (0.0183 mol) of EGDEMA, 0.11 g (106 mmol) of styrene and 0.096 g (0.201 mmol) of NHS-BlocBuilder are added to 5.12 g (4.95 mL) of 1,4 dioxane to the reactor and mixing with a magnetic stir bar started. After sealing the reactor and purging for 30 min with nitrogen, heating at 10 °C min^−1^ was commenced while maintaining the nitrogen purge. Samples were periodically withdrawn by syringe for molecular weight determination via GPC and conversion measurement by ^1^H NMR. For this case of EGDEMA/S-N-15–90, the reaction was completed after 120 min with an overall conversion of 0.71, styrene composition in the copolymer *F_S_* = 0.20, as measured with ^1^H NMR in CDCl_3_ and *M_n_* = 18.3 kg mol^−1^ and *Ð* = *M_w_/M_n_* = 1.67 according to GPC using poly(methyl methacrylate) standards in THF at 40 °C.

### 2.3. Chain Extension of Poly(EGDEA) with Styrene

The EGDEA homopolymer was used as a macroinitiator for subsequent chain extension with a fresh batch of styrene. The chain extension was performed using a 50 wt.% dioxane solution at 90 °C with *r* = 0.05. A formulation is given as an example (Expt. ID of macroinitiator: EGDEA in Table 2): SG1-terminated EGDEA (0.4209 g, 0.105 mmol), SG1 (0.0015 g, 0.005 mmol), styrene (4.3837 g, 42.090 mmol), and dioxane (4.3927 g, 49.855 mmol) were added to the reactor prior to being sealed with a rubber septum. The summary of the EGDEA homopolymer chain extension experimental conditions is found in Table 4. The mixture was purged with nitrogen for 30 min at room temperature prior to being heated at a rate 10 °C min^−1^. The time when the temperature reached 90 °C was arbitrarily taken as the start of the reaction (t = 0 min). The final polymer was recovered by precipitation in cold methanol followed by being dried in the vacuum oven for 6 hours at 25 °C. For the specific example cited, the polymerization was stopped after 2 h and the final fractionated block copolymer was characterized by a yield of 0.40 g with *M_n_* = 17 kg mol^−1^ and *Ð = M_w_/M_n_* = 1.34 according to GPC using poly(methyl methacrylate) standards in THF at 40 °C.

### 2.4. Chain Extensions from Poly(EGDEMA/S) with Styrene

The EGDEMA/S-N-15-90 (*M_n_* = 18.3 kg mol^−1^, *Đ* = 1.67, *F_S_* = 0.20) was used as a macroinitiator for subsequent chain extension with a fresh batch of styrene. The chain extension was performed using a 50 wt.% dioxane solution at 100 °C. The EGDEMA/S macroinitiator (2.01 g) and 2.00 g of styrene monomer were placed inside the 25 mL reactor under the same conditions as listed in previous sections along with dioxane (4.05 g). After the contents were added to the reactor, the reactor was sealed with a rubber septum and stirring started. The mixture was purged with nitrogen for 30 min at room temperature prior to being heated with a rate 10 °C min^−1^. The time when the temperature reached 90 °C was assumed to be the start of the reaction (*t* = 0 min). The final reactor set-point temperature was 100 °C. After 120 min, the polymerization was stopped by cooling the reactor. Once the reactor temperature was below 40 °C, the reactor was dissembled, and the polymer was precipitated into cold methanol followed by drying in the vacuum oven overnight at 25 °C. The final block copolymer had *M_n_* = 21.3 kg mol^−1^ and *Ð = M_w_/M_n_* = 1.72 according to GPC using poly(methyl methacrylate) standards in THF at 40 °C.

### 2.5. Chain Extensions from Poly(EGDEMA/S) with Butyl Methacrylate/Styrene

An EGDEMA/S macroinitiator (EGDEMA/S-N-80-90) with a lower EGDEMA content (*f_EGDEMA,0_* = 0.20) was prepared in 50 wt.% dioxane solution at 90 °C using the same protocols as in Section 2.2. Here, 0.0961 g of NHS-BB was added along with 3.0047 g of S, 1.8953 g of EGDEMA and 5.0792 g of dioxane and bubbled at room temperature with nitrogen for 30 min. This macroinitiator resulted in *M_n_* = 5.6 kg mol^−1^, *Đ* = 1.27, *F_EGDMA_* = 0.23 after 3 h polymerization at 90 °C (it was precipitated in methanol). For the chain extension, the macroinitiator (0.530 g) was added to a mixture of BMA (3.0020 g) and S (0.301 g) along with 3.805 g of dioxane solvent and bubbled under nitrogen for 30 min prior to raising the temperature to 90 °C. The chain extension was allowed to proceed for 2 h, after which the heating was stopped, and the polymer precipitated in methanol. The final chain-extended product had *M_n_* = 32.3 kg mol^−1^, *Đ* = 1.69 according to GPC relative to PMMA standards in THF and overall composition according to ^1^H NMR in CDCl_3_: *F_BMA_* = 0.64, *F_S_* = 0.31, *F_EGDEMA_* = 0.05.

### 2.6. Characterization Methods

#### 2.6.1. ^1^H NMR Spectroscopy

^1^H NMR spectra were recorded on a Varian Mercury 400 MHz spectrometer in CDCl_3_ and samples were scanned 32 times. Chemical shifts are reported in parts per million (ppm) using the residual of chloroform as the internal standard (7.26 ppm for ^1^H).

#### 2.6.2. Gel Permeation Chromatography

Molecular weight and dispersity of all polymers were characterized by GPC (Waters Breeze) using THF as the mobile phase at 40 °C in this study. The GPC was equipped with three Waters Styragel HR columns and a guard column. The molecular weight measurement ranges for the Styragel columns were: HR1: 10^2^ to 5 × 10^3^ g mol^−1^, HR2: 5 × 10^2^ to 2 × 10^4^ g mol^−1^, HR3: 5 × 10^3^ to 6 × 10^5^ g mol^−1^). The columns were operated at 40 °C and a mobile phase flow rate of 0.3 mL min^−1^ during analysis. The GPC had both an ultraviolet (UV 2487, Waters, Milford, MA, USA) and a differential refractive index (RI 2410, Waters, Milford, MA, USA) detector. The results reported were obtained from the RI detector. All molecular weight measurements were calibrated with poly(methyl methacrylate) narrow molecular weight distribution standards provided by Varian Canada (Mississauga, ON, Canada) (peak molecular weight (M_p_) range: 875–1,677,000 g mol^−1^).

## 3. Results and Discussion

**Figure 2:**
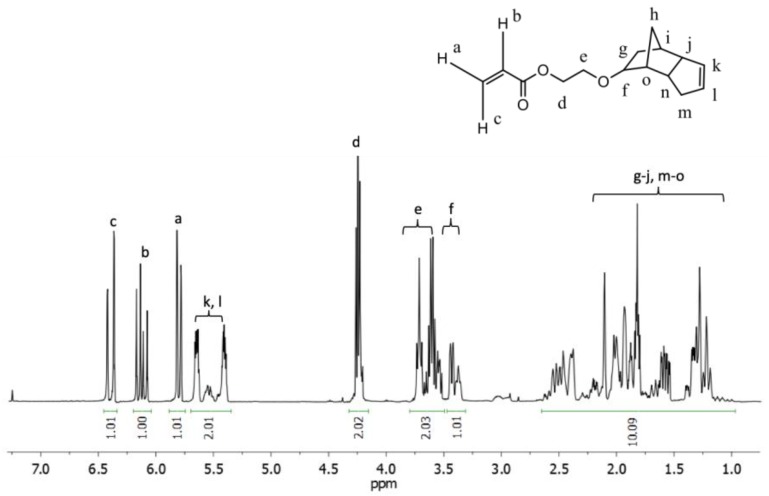
**^1^**H NMR of a sample of the reaction mixture for the homopolymerization of EGDEA (Expt. ID: E-T90, temperature = 90 °C, t_polymerization_ = 150 min, *M_n_* = 3.0 kg mol^−1^, *Ð* = 1.54).

A kinetic study of the effects of temperature and additional SG1 free nitroxide for the homopolymerization of EGDEA by NMP was conducted initially. ^1^H NMR spectroscopy was used to determine the monomer conversion. The 3 vinyl proton peaks of the EGDEA monomer are located at 5.80, 6.10 and 6.35 ppm. The two alkene protons of the cyclopentene moiety of EGDEA, shared by both the monomer and polymer, have peaks located between 5.35–5.65 ppm. The following equation was employed to calculate EGDEA monomer conversion by ^1^H NMR throughout the course of the reaction:

(1) $$\%\;monomer\;conversion=\frac{\left(m+p\right)-m}{\left(m+p\right)}\times 100\%$$

where (*m + p*) represents the integral area of the two alkene protons of the cyclopentenyl group present in both the monomer and polymer in the peak region 5.35–5.65 ppm (protons labeled *k, l*) and m represents the integral area of the vinyl proton present in only the monomer at 6.35 ppm (proton labeled *c*). For example, based on the ^1^H NMR spectrum displayed in Figure 2 below, the following sample calculation for conversion is:

(2) $$\%\;monomer\;conversion-=\frac{\left(\frac{2.35}{2}\right)-1.00}{\left(\frac{2.35}{2}\right)}\times 100\%=14.9\%$$

**Figure 3:**
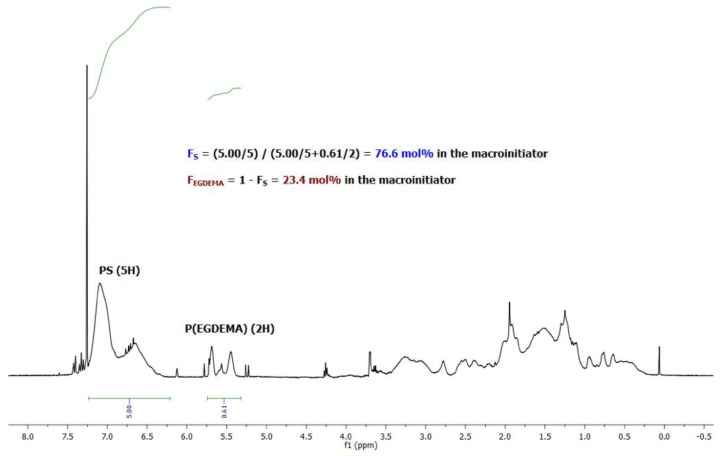
**^1^**H NMR of an EGDEMA/S copolymer (EGDEMA/S-N-80-90) with *M_n_* = 5.6 kg mol^−1^, *Ð* = 1.27, *F_S_* = 0.77. Note that the cyclopentenyl peaks at about 5.5 ppm were used to indicate EGDEMA composition while the aromatic protons at 6.5–7.0 ppm were used to indicate S composition.

A final copolymer ^1^H NMR is presented in Figure 3 for EGDEMA/S-N-80-90 (*M_n_* = 5.6 kg mol^−1^, *Đ* = 1.27, *F_EGDMA_* = 0.23) showing the removal of the vinyl groups from the monomers. Note that it was not possible to determine degree of polymerization by end-group analysis for this particular polymer due to overlapping peaks.

In comparison to vinyl aromatics (e.g., styrene), acrylates possess lower activation-deactivation equilibrium constants *K* in SG1-mediated systems [35,36,37], which strongly influences the control of the polymerization. TEMPO, as a mediator, was unsuccessful in controlling the homopolymerization of n-butyl acrylate [38] (a structurally comparable acrylate to EGDEA), due to the especially low value of *K*; e.g., *K* = 1.5 × 10^−13^ mol L^−1^ at 100 °C [36]. Selection of SG1 as the mediator instead of TEMPO increased the value of *K* (based on SG1 model by Lacroix-Desmazes et al. [39], *K* = 9.7 × 10^−12^ mol L^−1^ at 100 °C) and provided better control and much higher conversions of *n*-butyl acrylate [40,41]. Below, Equation (3) for the *K* of an NMP SG1-mediated system is defined where [P] is the concentration of active propagating radicals, [SG1] is the free SG1 concentration and finally [P-SG1] is the concentration of the dormant SG1-capped polymer. A higher value of *K* tends towards a system containing a high concentration of active propagating radicals thereby favoring irreversible termination [25]. This demonstrates why additional free SG1 nitroxide is added to the reaction; the equilibrium will shift to favour the dormant state. The effect of additional free nitroxide should allow a better control of the polymerization by NMP hence achieving relatively high molecular weights with active chain ends, while also keeping relatively low dispersity.

(3) $$K=\frac{\left[P\right]\left[SG1\right]}{\left[P-SG1\right]}$$

To gain a better understanding of the kinetics of an NMP-based system, the *k_p_K* values are often used as a tool characterizing the two main drivers: the propagation and equilibrium between dormant and active states [42]. Equation (4) below summarizes a relationship for *k_p_K* where *k_p_* denotes the average propagating rate constant and *K* is the equilibrium constant. Based on the relationship described in Equation (4), *k_p_K* values and their errors can be estimated by the slopes of the semi-logarithmic kinetic plots and standard errors in the slope, respectively.

(4) $$k_{p}K\approx k_{p}\left[P\cdot \right]\frac{{\left[SG1\cdot \right]}_{0}}{{\left[BlocBuilder\right]}_{0}}=k_{p}\left[P\cdot \right]r=\frac{d\left\{\ln{\left(1-X\right)}^{-1}\right\}}{dt}r$$

### 3.1. Effects of Temperature on EGDEA Homopolymerization Kinetics

**Figure 4:**
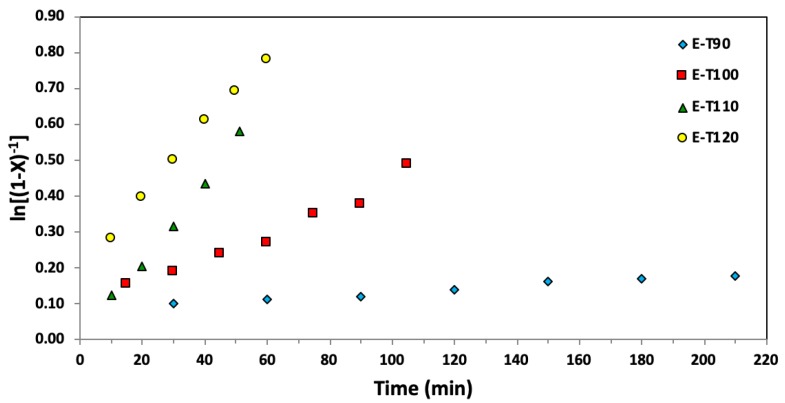
Semi-logarithmic kinetic plots of ln[(1−X)^−1^], where X represents the overall monomer conversion, versus time for ethylene glycol dicyclopentenyl ether acrylate (EGDEA) homopolymerizations at 90–120 °C via nitroxide mediated polymerization with BlocBuilder^TM^ and 5 mol% additional SG1 free nitroxide relative to BlocBuilder^TM^.

**Figure 5:**
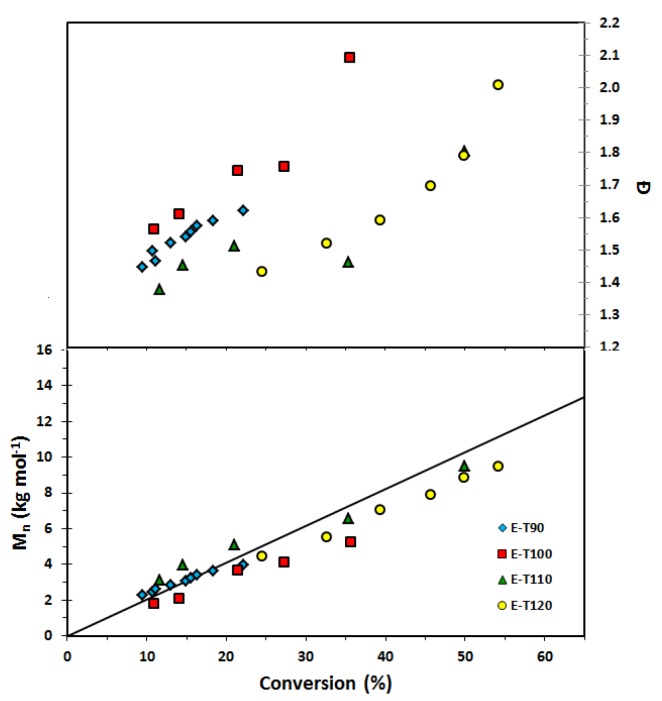
Number average molecular weight (*M_n_*) and dispersity (*Ð*) of homopolymers of ethylene glycol dicyclopentenyl ether acrylate (EGDEA) with *r* = 0.05 (added free SG1 nitroxide relative to BlocBuilder initially) at various temperatures (E-T90 = EGDEA homopolymerization at 90 °C; E-T100 = EGDEA homopolymerization at 100 °C; E-T110 = EGDEA homopolymerization at 110 °C; E-T120 = EGDEA homopolymerization at 120 °C). The straight solid line indicates the predicted *M_n_* versus conversion if the polymerization was living (M_n, theoretical_ at 100% conversion ≈ 20 kg mol^−1^ for this set of polymerizations).

Table 5 summarizes the EGDEA polymerizations (polymerization time, conversion and molecular weight distribution data) as a function of temperature at a fixed *r* = 0.05 while Table 6 lists the *k_p_K* values at various temperatures extracted from apparent rate constants associated with the semi-logarithmic kinetic plots of Figure 4. Based on the *k_p_K* values listed in Table 6 for EGDEA polymerizations, there is an observable trend of increasing *k_p_K* with temperature until 110 °C. At 120 °C, the *k_p_K* does not change appreciably from that at 110 °C, perhaps due to effects such as long chain branching caused by backbiting, which is common for acrylate polymerizations [40,43,44]. This is also reflected by the kinetic plots shown in Figure 4 where the intercept does not go through the origin for the points taken at 120 °C. Thus, extraction of *k_p_K* from the data at 120 °C is not reliable. Also, a review of controlled polymerizations suggested the K at higher temperature may lead to depolymerization [45], which was indicated in ATRP when adding inhibitors [46] and RAFT [47] systems. Additionally, it should be noted the *M_n_* and *Ð* versus conversion plots indicate continually broadening of the molecular weight distribution with conversion from the onset of the polymerization, as shown in Figure 5.

Compared to other acrylate homopolymerizations by NMP, the *k_p_K* for EGDEA is generally higher at comparable temperatures. For example, *k_p_K* = 1.9 × 10^−5^ s^−1^ for oligoethylene glycol acrylate (OEGA) at 115 °C [48] while for tert-butyl acrylate *k_p_K* = (3.4–7.6) × 10^−5^ s^−1^ (depending on solvent) at 115 °C [49] and (0.43–1.0) × 10^−6^ s^−1^ at 110 °C [50]. Butyl acrylate had *k_p_K =* 1.8 × 10^−5^ s^−1^ at 120 °C [35] while Couvreur et al. reported *k_p_K* = (1.1–1.8) × 10^−5^ s^−1^ at 120 °C for acrylic acid [51]. For hydroxyethyl acrylate, Bian and Cunningham reported *k_p_K* = (1.3–1.6) × 10^−6^ s^−1^ at 100 °C, 4.3 × 10^−6^ s^−1^ at 110 °C, and 2.6 × 10^−5^ s^−1^ at 120 °C using the related MONAMS initiator [42] while the same authors reported *k_p_K* = (3.6–4.6) × 10^−6^ s^−1^ at 112 °C and 9.9 × 10^−6^ s^−1^ at 120 °C in bulk for dimethylaminoethyl acrylate [52]. Thus, the *k_p_K* of EGDEA is comparable to other acrylates by NMP, but generally tends to be higher up to about 110 °C.

Overall, the addition of 5 mol% additional SG1 for all temperatures was not helpful in successfully controlling the homopolymerization of EGDEA by NMP as evidenced by the high *Ð*. The presence of side reactions of the propagating radicals, such as chain transfer and disproportionation, plays a more dominant role with conversion as the rate is not decreased to the same extent as propagation is by the nitroxide concentration accumulation through the persistent radical effect [53]. This would explain why the deviation becomes more prominent with higher conversions. It is worth noting that all final molecular weights were all below those from theoretical prediction. However, one must consider that our samples were calibrated against poly(MMA) standards and this accounts for some deviation of the molecular weights attained. Even increasing *r* to 0.10 resulted in better linearity of the semi-logarithmic kinetic plots but the *Ð* did not dramatically decrease with *Ð* ~1.6–1.7.

### 3.2. Effect of Additional SG1 Free Nitroxide on EGDEA Homopolymerization

**Figure 6:**
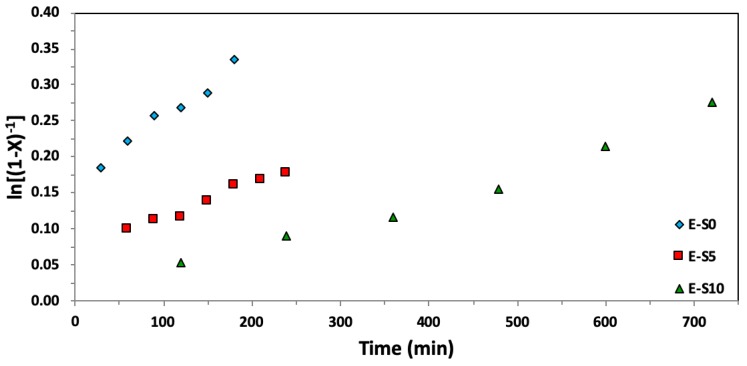
Semi-logarithmic kinetic plots of ln[(1−X)^−1^], where X represents the overall monomer conversion, versus time for ethylene glycol dicyclopentenyl ether acrylate (EGDEA) homopolymerizations at 90 °C via Nitroxide-Mediated Polymerization with BlocBuilder^TM^ and *r* = 0–10% excess SG1 free nitroxide relative to BlocBuilder^TM^.

**Figure 7:**
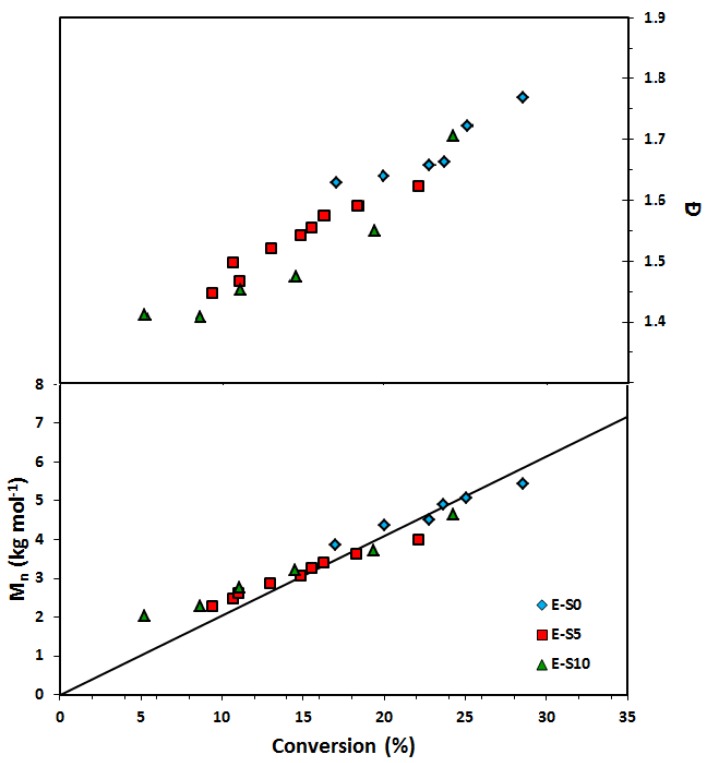
Number average molecular weight (*M_n_*) and dispersity (*Ð*) of homopolymers of ethylene glycol dicyclopentenyl ether acrylate (EGDEA) measured by GPC calibrated with poly(methyl methacrylate) standards (symbols: E-0 (*r* = 0): blue diamonds, E-5: red squares (*r* = 0.05), E-10: green triangles (*r* = 0.10)).

We subsequently continued our examination by fixing the polymerization temperature to one of the lower ones examined in the previous section: 90 °C. Based on Equation (3), increased concentrations of free SG1 nitroxide will effectively decrease the equilibrium constant *K* value and favor the dormant. This is due to a greater number of propagating radicals being temporarily capped by the additional free SG1 nitroxide. Therefore, it is reasonable to assume that the kinetics of the homopolymerization of EGDEA slows down with increasing mol % additional nitroxide. Figure 6 indicates some features that warrant attention. First, for lower concentrations of free nitroxide, the points do not go through the origin. One reason may be associated with the heating rate and the activity of the nitroxide, which is increased at temperatures increase beyond ~70 °C—this is near the lowest temperature, at which the nitroxide will begin dissociating [54]. However, with the heating rate being about 10 °C min^−1^, significant propagation would not be expected. Another plausible explanation is that there is insufficient nitroxide to keep the radicals in the dormant state, and thus there could be some curvature in the kinetic plots due to rapid propagation and termination events. At the highest level of free nitroxide studied (*r* = 0.10), the points pass near the origin, suggesting that the polymerization was relatively controlled at these conditions. Note that the conversions were kept relatively modest to avoid the gelation issues observed in earlier experiments. Further, despite the increased level of additional SG1 nitroxide, the homopolymerization of EGDEA was still marked by continuous broadening of the molecular weight distributions (see Figure 7) with final *Ð* = 1.62–1.76, as indicated in Table 5. Irreversible termination through disproportionation or chain transfer has likely occurred, rendering the SG1 nitroxide essentially ineffective in controlling the homopolymerization of EGDEA [55]. Another explanation could be that the hydroxylamine (SG1 nitroxide bonded to a hydrogen atom abstracted from propagating radical polymer chain) participates in the creation of dead polymer chains by transferring the hydrogen to cap another growing polymer chain [56]. The detailed measurement of long-chain branching is necessary in future optimization we are performing, particularly with respect to the acrylate, EGDEA.

### 3.3. Effect of Controlling Co-Monomer on EGDEA and EGDEMA Polymerizations

**Figure 8:**
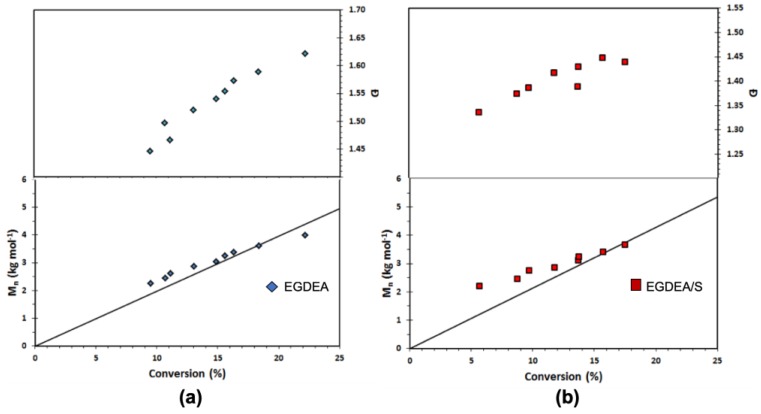
Number-average molecular weight (*M_n_*) and dispersity (**Ð**) versus conversion of (**a**) EGDEA homopolymerization and (**b**) EGDEA/S copolymerization with *f_S_*_,0_ = 0.05 in the initial monomer composition at 90 °C. This data is referred to expt. IDs EGDEA and EGDEA/S in Table 7.

**Figure 9:**
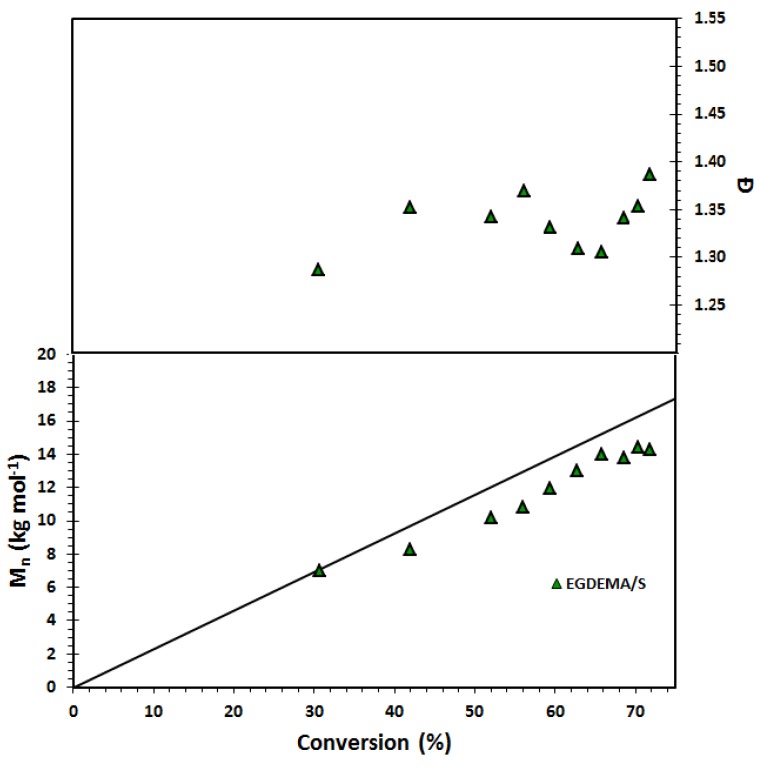
Number-average molecular weight (*M_n_*) and dispersity (*Ð*) of statistical copolymer of ethylene glycol dicyclopentenyl ether methacrylate (EGDEMA) and styrene (S) measured by GPC calibrated with poly(methyl methacrylate) standards (Expt. ID. EGDEMA/S in Table 7). This polymerization was done at 90 °C in 50 wt.% dioxane solution.

The controlling co-monomer concept with the BlocBuilder type of unimolecular alkoxyamine initiators has long been applied to control methacrylate-rich compositions [25]. Most controlling co-monomers have used styrenics, although acrylonitrile [57,58], and to some degree, isoprene has also been used [59]. While not strictly required for acrylates, based on the difficulties we had in keeping *Đ* low during the course of EGDEA homopolymerizations, we attempted the addition of small amounts of S, using *f_S_*_,0_ = 0.05. and *r* = 0.05. The results are summarized in Table 7 below and Figure 8. We polymerized to low conversion, comparable to the EGDEA homopolymerizations before the onset of gelation, but *Đ* was lower compared to the EGDEA homopolymerizations (1.44 versus 1.62 at nearly the same time and monomer conversion) and did not increase as severely during the polymerization. We next examined EGDEMA polymerizations controlled with *f_S_*_,0_ = 0.10 at 90 °C with *r* = 0.05 (a level typical for methacrylates with this alkoxyamine) and the results are plotted in Figure 9. We were able to polymerize to quite high conversions (72%) with fairly linear *M_n_* versus conversion plots together with nearly constant *Đ*, with the latter plateauing at about *Đ* = 1.38.

Encouraged by these results, we continued to examine EGDEMA/S polymerizations in more detail, as a function of temperature and initial molar composition of the co-monomers. In this last set, we also used the succinimidyl ester form of BlocBuilder, termed NHS-BlocBuilder, which effectively mimics the addition of free nitroxide since it dissociates at a rate 15 times faster than BlocBuilder [34]. This simplifies the process as only a single initiator is needed. Further, using NHS-BlocBuilder permits conjugation with other molecules in subsequent post-polymerization functionalization [60,61,62]. Table 8 summarizes the results for EGDEMA controlled by S with NHS-BlocBuilder at co-monomer initial mole fractions ranging from 0.05–0.15 and polymerization temperatures of 90 °C and 100 °C. As indicated in Table 8, the effect of controlling co-monomer concentration did not affect the conversion greatly and did not significantly narrow the molecular weight distribution. Temperature however did modulate the molecular weight distribution as at 100 °C, the *Ð* increased compared to those at 90 °C. Subsequently, polymerizations were suggested to be more effective at 90 °C.

**Figure 10:**
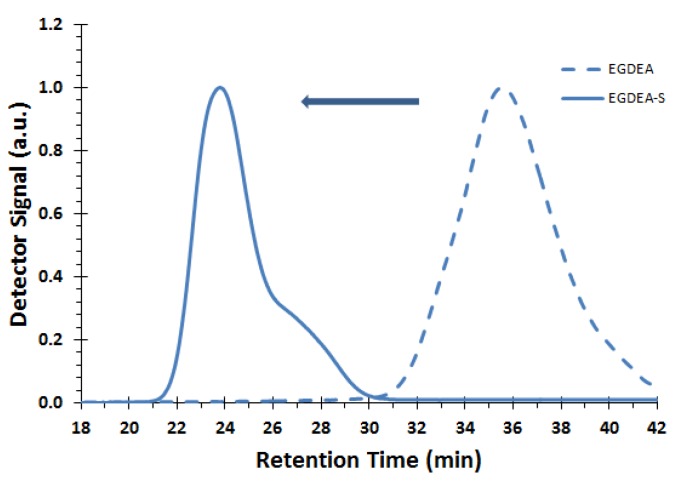
GPC traces of corresponding macroinitiator EGDEA (dashed line, sample E-T90, *M_n_* = 4.0 kg mol^−1^, *Ð* = 1.62) and chain extended block copolymer EGDEA-S (solid line, *M_n_* = 58.6 kg mol^−1^, *Ð* = 1.97, *F_EGDEA_* = 0.08). Chromatogram of chain-extended block copolymer showed no significant change after fractionation.

Chain extensions were performed using a macroinitiator to the EGDEA and the EGDEMA/S polymerizations (these were EGDEA homopolymer or EGDEMA-rich copolymer). Re-initiation of a chain with a fresh batch of monomer is often the most simple and direct way of assessing the chain end fidelity. We considered two chain extension reactions. The first one was using a homopolymer based on EGDEA (Expt. ID = E-T90, *M_n_* = 4.0 kg mol^−1^, *Ð* = 1.62), which was then chain-extended with a fresh batch of styrene at 100 °C. The GPC chromatogram of the macroinitiator and the chain-extended product is shown in Figure 10. After the chain-extension, a molecular weight increase was observed as evidenced by the shift to lower elution volumes in the GPC chromatograms. An overall broadening of the molecular weight distribution after the chain-extension was illustrated, suggesting the presence of further termination reactions taking place during the chain-extension. The small shoulder of the block copolymer EGDEA-S suggests possible bimolecular coupling occurring during the addition of the second block. However, there was no discernible peak associated with the macroinitiator, suggesting that it was sufficiently capped with SG1 groups to cleanly initiate the second block. While fractionation was performed on the crude block copolymer to remove species with the lower molecular weight, the GPC chromatogram characteristics were relatively unchanged. The increase in *Ð* was substantial, increasing from 1.62 to 1.97 for block copolymer EGDEA-S (*M_n_* = 58.6 kg mol^−1^), which was also very styrene rich (*F_EGDEA_* = 0.08 of the final polymer after fractionation). Thus, the bimodal product suggests that most chains were successful in being re-initiated and experiencing simultaneous chain growth, but some portion of the population was terminated during the chain extension.

**Figure 11:**
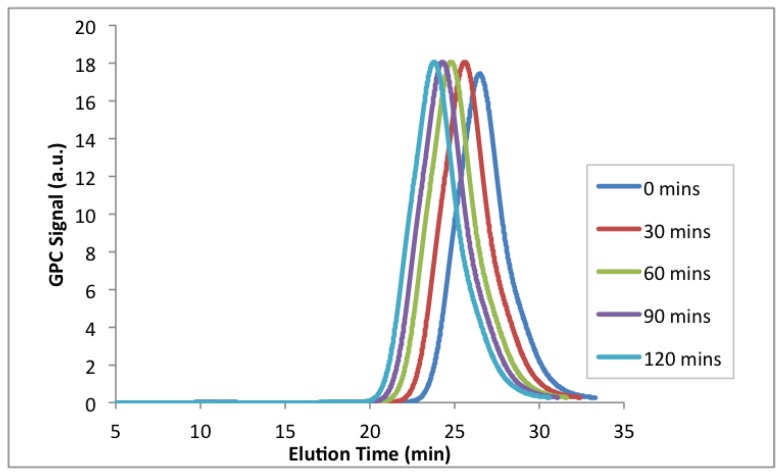
GPC chromatograms of the chain extension with styrene at 100 °C from a poly(EGDEMA-*stat*-S) macroinitiator (see Table 8 for properties of the macroinitiator with ID = EGDEMA/S-15-90).

The second chain extension using an EGDEMA-rich macroinitiator was performed using EGDEMA/S-N-15-90 as the macroinitiator (Table 8, *M_n_* = 18.3 kg mol^−1^, *Ð* = 1.67) for a fresh batch of styrene monomer at 100 °C. Figure 11 shows the GPC chromatograms for the chain extension taken from samples at various polymerization times. The peaks remained monomodal and progressively shifted to the left (higher molecular weights), indicating that the macroinitiator was sufficiently capped with active alkoxyamine end groups to permit block copolymer formation. The final chain extended copolymer after 120 min at 100 °C had *M_n_* = 21.3 kg mol^−1^ and *Ð* = 1.72, which is a modest increase but sufficient to indicate the retention of a monomodal molecular weight distribution.

**Figure 12:**
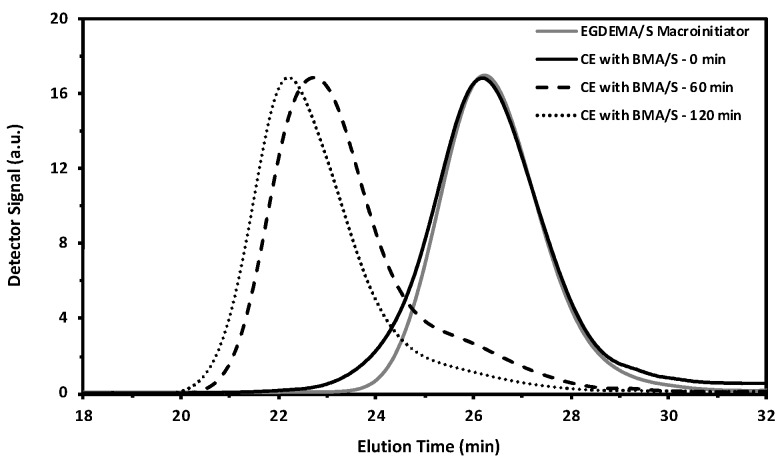
GPC traces from an EGDEMA/S macroinitiator (EGDEMA/S-N-80-90, *M_n_* = 5.6 kg mol^−1^, *Đ* = 1.27, *F_EGDEMA_* = 0.23) to the chain extended species with BMA/S at various polymerizations to form the poly(EGDEMA/S-block-BMA/S) block copolymer (*M_n_* = 32.3 kg mol^−1^, *Đ* = 1.69, *F_BMA_*= 0.64, *F_EGDEMA_* = 0.05).

**Figure 13:**
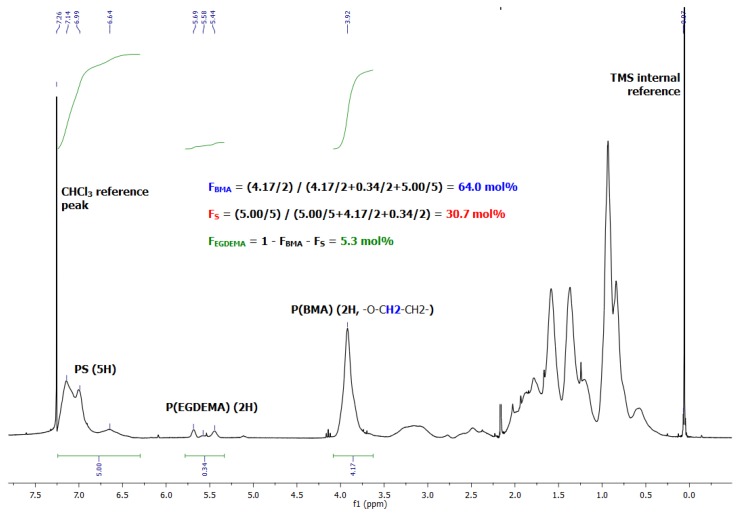
^1^H NMR spectrum in CDCl_3_ of poly(EGDEMA/S-block-BMA/S) showing the integrations to provide the overall compositions of the block copolymer.

We concluded our chain extension studies by using an EGDEMA/S macroinitiator that was much richer in S and was polymerized to fairly low conversion to ensure high chain end fidelity (*M_n_* = 5.6 kg mol^−1^, *Đ* = 1.27, *F_EGDEMA_* = 0.23). This was then chain-extended with a dissimilar monomer mixture of BMA/S (*f_BMA_*_,0_ = 0.90) batch of monomer to provide a more flexible second block. Such a block copolymer could be used as a template for thiol-ene reactions via modification of the EGDEMA units on the first block. The GPC traces are shown in Figure 12. The plots indicate steady growth to higher molecular weights with a small low molecular weight tail, indicating a low fraction of dead macroinitiator. The final chain-extended product possessed a broader molecular weight distribution but was nearly monomodal (*M_n_* = 32.3 kg mol^−1^, *Đ* = 1.69). The composition of the final recovered product was much richer in BMA as indicated in the ^1^H NMR spectra shown in Figure 13 (*F_BMA_* = 0.64, *F_S_* = 0.31). We are continuing this study by examining the modification of the EGDEMA-containing block to make it tougher, compared to the softer BMA-rich segment, a property that may be of value in thermoplastic elastomers for example.

## 4. Conclusions

EGDEA and EGDEMA polymerizations were performed using nitroxide mediated polymerization. The acrylate homopolymerization was studied as a function of temperature and additional SG1 free nitroxide. EGDEA was best controlled at lower temperature (90 °C) and *r* = 0.10 (concentration of free nitroxide to BlocBuilder initiator initially added), although dispersity *Đ* steadily increased (with final *Đ* ~ 1.6–1.7) during the course of the polymerization and gelation began being observed as conversions increased above 30%. Addition of a styrene (S) controlling co-monomer at initial mole fraction added *f_S_*_,0_ = 0.05 to the EGDEA formulation resulted in *Đ* not increasing as sharply during the polymerization with a lower *Đ* = 1.44 compared to the homopolymerization (*Đ* = 1.62). Chain extensions of the EGDEA/S macroinitiator indicated good chain fidelity with only some bimodal termination occurring during the chain extension. EGDEMA/S copolymerizations with *f_S_*_,0_ = 0.10 and *r* = 0.05 were controlled much better, with higher conversions obtained ~70%, with *Đ* = 1.38. Similar results were found for EGDEMA/S copolymerizations with *f_S_*_,0_ = 0.05–0.15 using a sole succinimidyl unimolecular initiator NHS-BlocBuilder, with relatively high conversions and good chain end fidelity, as witnessed by chain extensions with S monomer. However, *Đ* was somewhat higher (*Đ* = 1.56–1.67) compared to the system initiated with the BlocBuilder/SG1 pair. These results set up conditions to employ EGDEA and EGDEMA monomers into polymers with controlled architectures and possibilities for imparting additional functionality via post-polymerization orthogonal click chemistries. 

## Figures and Tables

**Table 1:** Experimental conditions for homopolymerization of ethylene glycol dicyclopentenyl ether acrylate (EGDEA) in 1,4-dioxane at 90–120 °C and various levels of free SG1 nitroxide via Nitroxide-Mediated Polymerization with BlocBuilder.

Expt. ID *^a^*	Temperature (°C)	[BlocBuilder]_0_ (mol L^−1^)	[SG1]_0_ (mol L^−1^)	*r ^b^*	[EGDEA]_0_ (mol L^−1^)	[1,4-dioxane]_0_ (mol L^−1^)	*M*_*n*,target_*^c^* (kg mol^−1^)
E-T90 (E-S5)	90	0.026	0.001	0.05	2.01	5.68	21.2
E-T100	100	0.024	0.001	0.06	1.98	5.76	20.4
E-T110	110	0.024	0.001	0.06	2.01	5.69	20.8
E-T120	120	0.024	0.001	0.06	2.01	5.68	21.1
E-S0	90	0.024	0.000	0	2.02	5.67	21.1
E-S10	90	0.025	0.003	0.10	2.11	5.93	21.3

*^a^* The Expt. ID E-TXX denotes the monomer used in homopolymerization (E for EGDEA) and XX represents the temperature in [=] °C used for the polymerization. The Expt. ID E-SX denotes the monomer used in homopolymerization (E for EGDEA) and SX represents the % excess free SG1 nitroxide relative to BlocBuilder. *^b^ r* = [SG1]_0_/[BlocBuilder]_0_ *^c^* Number average molecular weight at 100% conversion.

**Table 2:** Experimental conditions for ethylene glycol dicyclopentenyl ether acrylate/styrene (EGDEA/S) and ethylene glycol dicyclopentenyl ether methacrylate/styrene (EGDEMA/S) mixtures polymerized at 90 °C in 50 wt.% 1,4-dioxane solution with BlocBuilder^TM^ and additional SG1 free nitroxide.

Expt. ID *^a^*	[BlocBuilder]_0_ (mol L^−1^)	[SG1]_0_ (mol L^−1^)	*r ^b^*	[Styrene]_0_ (mol L^−1^)	[X]_0_ *^c^* (mol L^−1^)	[1,4-dioxane]_0_ (mol L^−1^)	*M*_*n*,target_*^d^* (kg mol^−1^)
EGDEA/S	0.024	0.001	0.051	0.11	1.99	5.61	21.5
EGDEMA/S	0.023	0.001	0.051	0.21	1.91	5.41	23.1
EGDEA (E-T90)	0.026	0.001	0.053	0	2.01	5.68	21.2

*^a^* Expt. ID refers to the monomer (EGDEA or EGDEMA) either being statistically copolymerized in the small presence of a known controlling comonomer (styrene denoted as S) or homopolymerized. *^b^ r* = [SG1]_0_/[BlocBuilder^TM^]_0_. *^c^* X denotes the monomer (EGDEA or EGDEMA) used for the corresponding Expt ID. *^d^* Number average molecular weight at 100 % conversion.

**Table 3:** Experimental conditions for ethylene glycol dicyclopentenyl ether methacrylate/styrene (EGDEMA/S) mixtures polymerized in 50 wt.% 1,4-dioxane solution with NHS-BlocBuilder.

Experiment ID	T (°C)	[NHS-BB]_0_ (mol L^−1^)	[EGDEMA]_0_ (mol L^−1^)	[Styrene]_0_ (mol L^−1^)	*f*_*S*,0_	Reaction Time (min)
EGDEMA/S–N-5-90	90	0.022	1.93	0.10	0.05	90
EGDEMA/S–N-10-90	90	0.022	1.89	0.21	0.10	90
EGDEMA/S–N-15-90	90	0.022	1.84	0.33	0.15	120
EGDEMA/S-N-5-100	100	0.022	1.95	0.10	0.05	90
EGDEMA/S-N–10-100	100	0.022	1.90	0.21	0.10	90
EGDEMA/S–N-15-100	100	0.022	1.85	0.33	0.15	90
EGDEMA/S-N-80-90	90	0.020	0.71	2.86	0.80	150

**Table 4:** Experimental conditions for chain extension experiment with styrene from a poly(ethylene glycol dicyclopentenyl ether acrylate) macroinitiator at 90 °C.

Expt. ID *^a^*	[Macroinitiator]_0_ *^b^* (mol L^−1^)	[Styrene]_0_ (mol L^−1^)	[SG1]_0_ (mol L^−1^)	[1,4-dioxane]_0_ (mol L^−1^)	*M*_*n*,target_*^c^* (kg mol^−1^)
EGDEA-S	0.012	4.79	0.0006	5.68	45.7

*^a^* Expt. ID refers to the SG1-terminated homopolymer of EGDEA being chain extended with styrene (S) to form an EGDEA-S block copolymer. *^b^* The macroinitiator is SG1-terminated poly(EGDEA); Expt. ID. EGDEA from Table 2, *M_n_* = 4.0 kg mol^−1^, *Ð* = 1.62. *^c^* Number average molecular weight at 100% conversion.

**Table 5:** Summary of polymerizations and molecular properties for the homopolymerizations of ethylene glycol dicyclopentenyl ether acrylate (EGDEA) via nitroxide-mediated polymerization with BlocBuilder^TM^ and 5 mol% excess free SG1 nitroxide relative to BlocBuilder^TM^ in 1,4-dioxane at 90–120 °C.

Expt. ID *^a^*	Temperature (°C)	*r ^b^*	Polymerization Time (min)	Conversion *^c^* (%)	*M*_*n*,target_ (kg mol^−1^)	*M_n_^d^* (kg mol^−1^)	*Ð* *^d^*
E-T90 (E-S5)	90	0.05	270	22	19.8	4.0	1.62
E-T100	100	0.06	100	36	20.7	5.2	2.09
E-T110	110	0.06	50	51	20.8	9.5	1.80
E-T120	120	0.06	60	54	21.1	9.4	2.01
E-S0	90	0.00	180	29	21.1	5.4	1.76
E-S10	90	0.10	720	24	20.7	4.6	1.70

*^a^* The experiment ID E-TX denotes the temperature used in the homopolymerization of EGDEA (T for temperature) and X represents the sample’s reaction temperature in °C. The experiment ID E-SX denotes the mol % excess free SG1 nitroxide relative to BlocBuilder^TM^ used in the homopolymerization of EGDEA (S for SG1) and X represents the %. *^b^ r* = [SG1]_0_/[BlocBuilder]_0_. *^c^* Final conversion determined by ^1^H NMR. *^d^* Number average molecular weight and dispersity *Ð* of the homopolymer measured by GPC relative to poly(methyl methacrylate) standards in THF at 40 °C.

**Table 6:** Summary of *k_p_K* values for the EGDEA homopolymerization via NMP with BlocBuilder^TM^ in the presence of 5 mol % excess SG1 free nitroxide relative to BlocBuilder^TM^.

Temperature (°C)	*k_p_K* (10^−5^ s^−1^)
90	1.0 ± 0.1
100	6.8 ± 0.7
110	18.8 ± 1.0
120	16.7 ± 0.5

**Table 7:** Summary of polymerizations and molecular properties for the statistical copolymerizations ethylene glycol dicyclopentenyl ether acrylate (EGDEA) and ethylene glycol dicyclopentenyl ether methacrylate (EGDEMA) with styrene and homopolymerization of EGDEA via Nitroxide-Mediated Polymerization with BlocBuilder^TM^ in 1,4-dioxane at 90 °C.

Expt. ID *^a^*	T (°C)	*f_S_*_,0_ *^b^*	Polymerization Time (min)	X *^c^*	*F_S_* *^b^*	<*k_p_*><*K*> (10^−6^ s^−1^)	*M_n_*_,target_ (kg mol^−1^)	*M_n_^d^* (kg mol^−1^)	*Ð ^e^*
EGDEA/S	90	0.05	320	0.17	0.14	9.5 ± 0.9	21.5	3.6	1.44
EGDEMA/S	90	0.10	130	0.72	0.13	7.0 ± 0.5	23.1	14.3	1.38
EGDEA	90	0	270	0.22	0	145 ± 4	19.8	4.0	1.62

*^a^* Expt. ID refers to the statistical copolymerizations of ethylene glycol dicyclopentenyl ether acrylate (EGDEA) or ethylene glycol dicyclopentenyl ether methacrylate (EGDEMA) with styrene (S) or the homopolymerization of EGDEA. *^b^* Initial feed composition and final copolymer composition relative to S measured by ^1^H NMR. *^c^* Overall monomer conversion X determined by ^1^H NMR. *^d^* $\langle k_{p}\rangle \langle K\rangle$ values were derived from the apparent rate constants from the ln[(1−X)^−1^] versus polymerization time plots that were used to estimate the product of the average propagation rate constant $\langle k_{p}\rangle$ and equilibrium constant between dormant and active chains 〈K〉. *^e^* Number average molecular weight *M_n_* and dispersity *Ð* of either the copolymer or homopolymer relative to poly(methyl methacrylate) standards in THF at 40 °C.

**Table 8:** Summary of EGDEMA-rich polymerizations controlled with styrene (S) at 90 and 100 °C using NHS-BlocBuilder in 50 wt.% 1,4 dioxane solutions.

Experiment ID *^a^*	Temperature (°C)	*t* (min)	*f_S_*_,0_ *^b^*	*F_S_* *^b^*	X *^c^*	*M_n_* (kg mol^−1^)^d^	Ð *^d^*
EGDEMA/S–N-5-90	90	90	0.05	0.08	0.64	13.2	1.64
EGDEMA/S-N–10-90	90	90	0.10	0.14	0.65	15.8	1.56
EGDEMA/S-N–15-90	90	120	0.15	0.20	0.71	18.3	1.67
EGDEMA/S-N–5-100	100	90	0.05	0.07	0.69	17.3	1.73
EGDEMA/S–N-10-100	100	90	0.10	0.13	0.71	21.9	1.76
EGDEMA/S–N-15-100	100	90	0.15	0.17	0.67	16.4	1.74
EGDEMA/S-N-80-90	90	150	0.80	0.77	0.30	5.6	1.27

*^a^* Expt. ID refers to the statistical copolymerizations of ethylene glycol dicyclopentenyl ether methacrylate (EGDEMA) with styrene (S) with EGDEMA/S-N-xx-yy referring to copolymerizations with N = NHS-BlocBuilder, xx = % initial mole fraction of S in monomer feed and yy = temperature (^o^C) of polymerization. *^b^* Initial feed composition of styrene = *f_S_*_,0_ and final copolymer composition relative to S is *F_S_*, which is measured by ^1^H NMR. *^c^* Overall monomer conversion X determined by ^1^H NMR. *^d^* Number average molecular weight and dispersity *Ð* of either the copolymer or homopolymer relative to poly(methyl methacrylate) standards in THF at 40 °C.
